# Medical induction of maternal behavior in nurse mares and foal-rejecting mares undergoing foal grafting: a retrospective study of 140 cases

**DOI:** 10.1093/jvimsj/aalag131

**Published:** 2026-07-06

**Authors:** Linnéa M Birgitsdotter, Hanna Friedrichsen Borg, Anna Kendall

**Affiliations:** Distriktsveterinärerna Stenungsund, Ödsmål, Sweden; Distriktsveterinärerna Stenungsund, Ödsmål, Sweden; Mälaren Equine Clinic, Sigtuna, Sweden

**Keywords:** adoption, horse, orphan, oxytocin, prostaglandin

## Abstract

**Background:**

Medical induction of maternal behavior can be a safer and less stressful way to introduce orphan foals to nurse mares, compared with traditional methods that are time consuming and require restraint of the mare. Published data on results of medical treatment in a large number of mares are lacking.

**Hypothesis/Objectives:**

Describe a hormone-based protocol for foal grafting, including nonsteroidal anti-inflammatory drugs, dinoprost tromethamine, and oxytocin, and to report treatment outcome.

**Animals:**

A total of 113 nurse mares and 27 mares that had rejected their foals.

**Methods:**

Retrospective case series. Cases were retrieved through a record search from a nation-wide veterinary field practice and from one equine referral hospital in Sweden.

**Results:**

Overall, 71% of the cases had a successful outcome. In most successful cases, acceptance was immediate with minimal restraint of the mare required. Mares that rejected their foals at birth had a worse treatment outcome (52% successful) than nurse mares (76% successful).

**Conclusions and clinical importance:**

The foal-grafting protocol was a time-efficient, safe, and effective alternative to traditional methods. Considering the decreased need for restraint of the mare it also has animal welfare benefits. No adverse effects other than profuse sweating were reported in the treated mares.

## Introduction

Orphan foal management presents a substantial practical challenge. Hand-rearing is possible but labor-intense, costly, and may lead to behavioral abnormalities if an appropriate social environment is not provided.^1^ Using a nurse mare is often the most practical solution, with the ideal nurse mare considered to be a peripartum mare in full lactation.^2^ Successful induction of lactation and adoption by non-parturient mares also has been reported.^3^^,^^4^ Traditional foal adoption approaches rely on physical and medical restraint of the mare during introduction. These approaches are time-consuming, potentially hazardous for both foal and handler, and may take several days.^5^ Vagocervical stimulation (VCS), mimicking stimuli experienced during parturition, has been shown to increase acceptance rates and to decrease acceptance time in mares and ewes,^4^^,^^6^ but does not exclude the need for restraint. Additional techniques include masking the scent of the foal and stopping the mare from sniffing the foal, because olfactory cues play a crucial role in offspring recognition.^7–10^

The establishment of normal maternal behavior is a complex process influenced by multiple hormones released during parturition, in combination with olfactory cues and visual exposure to the neonate during a short and critical postpartum period.^10^ The relative contributions of specific hormones to maternal behavior remain incompletely understood and vary among species.^11^ During the second stage of parturition in the horse, prostaglandins and oxytocin play pivotal roles by inducing strong uterine contractions that expel the fetus.^12^ Experimental evidence from other species indicates that central oxytocin administration induces maternal behaviors,^13–15^ suggesting a central neurobiological mechanism for maternal acceptance. Prostaglandins also promote maternal behavior across multiple species, potentially through stimulation of central oxytocin release.^16–19^ Prostaglandin F2α has been used clinically to induce maternal behavior in mares, but evidence remains sparse and treatment protocols vary.^2^^,^^3^^,^^20^ Artificially replicating the endogenous periparturient hormonal cascade may promote maternal behavior in adoption scenarios. A hormone-based protocol combining flunixin meglumine, dinoprost tromethamine, and oxytocin is used in clinical practice, but treatment efficacy has only been documented in a single case report.^21^

We hypothesized that this hormone-based protocol would effectively and rapidly induce maternal behavior and promote orphan foal acceptance in nurse mares, and that the protocol also would be effective in cases of maternal rejection. Our study describes a medical foal-grafting protocol and reports the treatment results in nurse mares and foal rejection cases.

## Materials and methods

The study was conducted as a retrospective case series. Clinical records were obtained from Distriktsveterinärerna, a nationwide general field practice in Sweden, and Mälaren Hästklinik, an equine referral hospital. A record search was conducted for horses treated with dinoprost tromethamine between 2015 and 2025 or with a diagnosis of hormonal treatment for foal grafting. Records were manually reviewed for treatments of foal grafting and included if the protocol described below was used. Horses were excluded if records lacked documentation of treatments or outcome, or if the mare received additional treatments (eg, VCS) that could influence the outcome. Data included mare age, foal age, mare breed, reproductive history, case type (foal rejection: mares that did not accept their own foal after foaling; nurse mare: mares without a nursing foal of their own that were introduced to an orphan foal), history of foal loss before fostering, treating veterinarian, treatments (including dosage and repeated treatments), and outcome.

Initially, foal and mare were placed in separate stalls with no visual contact. The mare received a nonsteroidal anti-inflammatory drug (NSAID) IV. After 30 min, dinoprost tromethamine was administered IM in the neck. As the mare subsequently began sweating, the sweat was collected and rubbed onto the foal. After an additional 15-20 min, the mare was injected with oxytocin IV. After a few minutes, the foal was introduced. The mare was allowed to sniff and examine the foal at this point. If the mare did not show immediate acceptance of the foal, oxytocin administration was repeated at the same dosage as previously used.

Foal acceptance was defined as the mare displaying maternal behaviors such as licking, nickering, and sniffing the foal and subsequently letting the foal nurse. Rejection was defined as aggressive behavior, ignoring the foal and not allowing nursing.

Statistical analysis was performed using Jamovi (version 2.6.44; The Jamovi project). Descriptive statistics, including means and SDs for continuous variables (eg, age), frequencies, and percentages for categorical variables (eg, breed, outcome) were calculated. Associations between treatment outcome (success/failure) and mare/foal demographics were evaluated using chi-squared or Fisher’s exact tests (expected cell counts < 5), and binomial logistic regression models. Odds ratios (ORs) with 95% CIs were reported, and statistical significance was set at *P* < .05. Cases with missing data were excluded from specific analysis in given subgroup analyses. Breeds with low representation were grouped into “other breeds” to ensure model stability. For parity analysis in the nurse mare group, mares were classified as nulliparous or parous (having had ≥ 1 foal).

## Results

### Horses

A total of 140 cases met the inclusion criteria (144 treatments). The cases were divided into a nurse mare group (113 cases, 115 treatments) and a foal rejection group (27 cases, 29 treatments). Demographic data are presented in Table 1. There were some missing data regarding breed, age, and previous foaling history. Seventeen different breeds were represented in the nurse mare group, and eight different breeds in the foal rejection group. Breed distribution reflected the general horse population in Sweden.^22^ The nurse mare group included both lactating and non-lactating individuals, mares that recently lost their foals, and mares that had just weaned their foals.

**Table 1:** Demographics and clinical characteristics of nurse mares, rejecting mares, and associated foals.

**Variable**	**Category**	**Nurse mares *n* = 113**	**Rejecting mares *n* = 27**
**Mare age (years)** ^**a**^	Mean (±SD)	13 (±5)	8 (±5)
**Foal age (days)** ^**b**^	Median (range)	3 (0-105)	1 (0-13)
**Breed** ^**c**^	Standardbred	22 (29)	3 (13)
	Warmblood	18 (24)	6 (26)
	NSH	11 (14)	3 (13)
	Arabian	1 (1)^g^	5 (22)
	Shetland pony	2 (3)^g^	3 (13)
	Other breeds	22 (29)	3 (13)
**Parity** ^**d**^	Nulliparous	3 (3)	n/a
	Primiparous	14 (14)	17 (63)
	Multiparous	37 (37)	4 (15)
	Unspecified	47 (47)	6 (22)
**Foaled current year** ^**e**^	Yes	58 (59)	n/a
	No	41 (41)	n/a
**Time since foal death (days)** ^**f**^	Median (range)	3 (0-60)	n/a

Data are presented as absolute numbers (%) for categorical variables, and as mean ± SD (if normal data) or median with range (if non-normal data) for continuous variables.

a Mare age available for 68/113 nurse mares and 19/27 rejecting mares.

b Foal age available for 85/113 foster foals and 24/27 rejected foals.

c Breed reported for 76 nurse mares and 23 rejecting mares.

d Reproductive status reported for 101 nurse mares and 21 rejecting mares. Unspecified mares were at a least primiparous, but total number of offspring was not reported.

e Foaling during current year reported for 99 nurse mares.

f Time since foal death reported for 55 of the 57 nurse mares that recently lost their foal.

g Included in “other breeds” in statistical analyses.

Abbreviations: NSH = North Swedish horse; n/a = not applicable.

### Treatments

Because of the retrospective nature of the study, some variations in treatment occurred.

Weight was estimated for horses seen in ambulatory practice (*n* = 133) whereas hospitalized mares were weighed (*n* = 7). All mares received an NSAID IV before hormonal treatment. A total of 135 mares received flunixin meglumine (1.1 mg/kg) and 9 mares received meloxicam (0.6 mg/kg).

All mares were treated with dinoprost tromethamine IM (5 mg/mL, Dinolytic; Zoetis, Kalamazoo, MI, USA). Most mares received a single dose of 10 mg. Ten mares received variable doses between 7.5 and 25 mg. In 2 mares, an additional dose was administered because of an insufficient sweating response after the initial injection; 1 mare received 10 mg followed by an additional 10 mg 40 min later and 1 mare received 10 mg followed by 5 mg 15 min later.

All mares were treated with oxytocin after the dinoprost tromethamine. A total of 118 mares received a single dose of 20-30 IU oxytocin IV. Eight mares received the same dose IM. Two mares received a single dose of 50 IU IV, 1 mare received 10 IU IV, and 1 mare received 5 IU IV. Fourteen of the mares that were given an initial dose of 20-30 IU IV received a second dose of oxytocin because of a perceived insufficient response (5, IM; 9, IV).

In 5 cases, a twitch was used for restraint when the mare and foal were introduced (2 successful outcomes, 3 failures), and 7 mares received light sedation with detomidine and butorphanol tartrate IV at introduction (4 successful, 3 failures). In 1 mare, dinoprost tromethamine treatment yielded an unsatisfactory sweating response, and the foal instead was covered in liquid obtained from uterine lavage (successful).

Treatments were administered by 107 different veterinarians, and the description of foal introduction was neither standardized nor consistently recorded in detail in the clinical records. The method therefore may have varied slightly, but generally involved minimal physical restraint and beginning with the mare being allowed nose contact with the foal.

### Treatment outcome

In general, the mares accepted or rejected the foal immediately after treatment. In 2 mares, the acceptance was delayed for up to 2 h, and in another 2, full acceptance was achieved within 24 h. Treatment outcomes are presented in Table 2.

**Table 2:** Treatment outcomes presented as total number (%).

**Treatment outcome**	**All mares**	**Nurse mares (*n* = 113)**	**Rejecting mares (*n* = 27)**
**Successful cases**	100/140 (71)	86/113 (76)	14/27 (52)
**Successful treatments**	100/144 (69)	86/115 (75)	14/29 (48)
**Successful, single dose OX**	94/130 (72)	80/104 (77)	14/26 (54)
**Successful, repeated dose OX**	6/14 (43)	6/11 (55)	0/3 (0)

“Treatment” defined as the whole treatment protocol including NSAID, dinoprost tromethamine, and oxytocin.

Abbreviation: OX oxytocin.

The odds of mare acceptance of the foal were significantly lower when repeated doses of oxytocin were needed compared with mares needing only a single dose (OR, 0.29; 95% CI, 0.09-0.89; *P* = .03).

#### Treatment outcome for nurse mares

Mare age was not significantly associated with treatment outcome (OR, 0.99; 95% CI, 0.89-1.10; *P* = .85). In contrast, foal age was significantly associated with treatment outcome, with increasing foal age associated with decreased odds of a successful outcome (OR, 0.94 per day of age; 95% CI, 0.91-0.98; *P* = .003).

No association was detected between breed and treatment outcome (*P* = .5).

Foal loss during the current year was not significantly associated with treatment outcome (OR, 1.85; 95% CI, 0.68-5.01; *P* = .22). Time elapsed since foal death was not associated with treatment outcome (OR, 1.04 per day; 95% CI, 0.96-1.12; *P* = .37).

When reproductive history was dichotomized into nulliparous and parous mares, nulliparous mares had higher odds of a negative treatment outcome compared with parous mares, but this difference was not significant (OR, 6.9; 95% CI, 0.6-79.8; *P* = .14).

#### Treatment outcome for foal rejection mares

The proportion of mares accepting their own rejected foal was significantly lower than the proportion of mares accepting foster foals (OR, 0.34; 95% CI, 0.14-0.81; *P* = .01).

Mare age was not significantly associated with treatment outcome (OR, 1.08; 95% CI, 0.87-1.34; *P* = .48). Also, foal age was not significantly associated with treatment outcome (OR, 0.52; 95% CI, 0.23-1.19; *P* = .12).

No association was detected between breed and treatment outcome (*P* = .88). Treatment outcome did not differ between primiparous and multiparous mares (*P* = 1.00), with similar odds of a positive outcome between groups (OR, 0.70; 95% CI, 0.08-6.22).

## Discussion

Our study describes a protocol for medical induction of maternal behavior in nurse mares and mares rejecting their foals. In this retrospective case series, the protocol achieved an overall success rate of 71%, with immediate acceptance without the need for restraint or tranquilization of the mare in most cases. This result is a marked improvement over traditional foal-grafting techniques, which encompass restraint, disciplinary handling, tranquilization or some combination of these, and has a reported acceptance time of up to several days.^4^^,^^23^

A few hormonal foal-grafting protocols have been described previously in the veterinary literature. One textbook suggests administration of 3-4 times the luteolytic dose of prostaglandin as a single component, preferably cloprostenol sodium (750-1000 μg).^2^ This protocol was evaluated in an abstract^20^ including 17 foster mares and 4 cases of foal rejection, with a reported overall success rate of 95%. However, no full publication is available for detailed evaluation. Also, a single-case report describes an approach using 25 mg dinoprost tromethamine IM, followed by 500 μg cloprostenol sodium when immediate acceptance did not occur.^3^

The prostaglandin dosages used in our study were lower than those previously described,^2^^,^^3^^,^^20^ but nearly all mares exhibited anticipated adverse effects such as sweating and restlessness—signs identified as crucial for initiating maternal behavior.^2^ This observation suggests that a 10 mg dinoprost tromethamine dose is sufficient. Dosing was individualized rather than adjusted for body weight, and the limited number of horses receiving higher doses precluded assessment of a potential dose–response relationship. Studies in other species have identified dose-dependent effects between prostaglandins and the onset and duration of maternal and parturition-related behaviors,^16^^,^^24^ indicating that higher doses potentially could influence treatment efficacy. Prostaglandins possess a wide safety margin, with doses of up to 200 mg/day deemed safe. High doses result in numerous adverse effects, but these are transient.^25^

The precise mechanism by which prostaglandins induce maternal behavior remains incompletely understood.^3^ The immediate behavioral response observed in most cases in our study supports a clinically relevant effect of dinoprost tromethamine. Both dinoprost tromethamine and its synthetic analog, cloprostenol sodium, appear capable of inducing maternal behavior.^3^^,^^20^ Cloprostenol sodium has a stronger luteolytic effect and fewer smooth muscle contraction adverse effects than dinoprost tromethamine, allowing for lower dosages.^26^ Research in rats and pigs has indicated that dinoprost tromethamine may exert central effects by crossing the blood–brain barrier,^17–19^^,^^27^ whereas the effect of cloprostenol is predominantly peripheral and has failed to induce maternal behavior in experimental settings.^19^ It is unknown whether central or peripheral activity predominates in maternal behavior in mares, and whether choice of prostaglandin influences treatment outcome.

The 30-min delay between dinoprost tromethamine injection and foal introduction in the currently described protocol may not be ideal. The drug has rapid absorption and elimination^28^ and may not have had maximum physiological impact at the moment of introduction. Although the effects on smooth muscle can persist for hours,^29^ the acute central effects relevant to maternal behavior induction are likely time-sensitive.^18^ The delay for sweat collection and oxytocin injection may need to be adjusted for optimization of overall efficacy. The method to collect and apply sweat from the mare onto the foal, leveraging the prostaglandin-induced diaphoresis,^25^ aligns with the known importance of olfactory cues in maternal recognition.^7–9^^,^^30^ Other hormonal protocols do not mention scent alteration,^2–4^ and it is not known whether this step is essential for successful foal grafting during hormonal induction. Until proven otherwise, continuing the use of olfactory masking in the protocol can be a straightforward way to increase the chances of successful adoption.

Flunixin meglumine is included in the protocol primarily for its analgesic properties, with the aim to mitigate prostaglandin-induced discomfort. However, its use also presents a potential pharmacological paradox. As a cyclooxygenase inhibitor, flunixin inhibits prostaglandin synthesis^31^ and could theoretically counteract aspects of the multi-hormone protocol.^32^ The need for NSAID administration as well as any disadvantageous interaction of the drug remains unclear.

Oxytocin is considered a key hormone in the maternal part of the maternal–neonate bonding process.^33^ Although equine-specific data are limited, studies in other species have demonstrated that oxytocin can induce maternal behaviors through central mechanisms.^13–15^ However, the behavioral effects of peripheral administration have been inconsistent,^2^^,^^13^^,^^32^^,^^34^^,^^35^ possibly because of limited transport across the blood–brain barrier.^35–37^ In our study, no association was detected between oxytocin dose and treatment outcome within the administered range. Mares receiving repeated oxytocin administrations had lower odds of a successful outcome compared with mares treated with a single dose. Repeated dosing was implemented in cases that did not respond adequately to the initial treatment and therefore may reflect individual lack of normal hormonal responses. There may be a difference between central and peripheral effects of oxytocin, and the specific contribution of peripheral oxytocin administration to treatment success is unknown. In addition to potential central effects, IV oxytocin stimulates milk letdown,^38^ which is likely to be advantageous during foal grafting.

Vaginocervical stimulation, consisting of manual stimulation of the cervix and vagina for two 2-min intervals, also has been described as an aid for foal grafting and was reported to enhance acceptance rates in 8 mares.^4^ In the record search, 7 mares were identified that received the hormonal protocol in combination with VCS, 6 of which were successful. These cases were all excluded from our study population to avoid potential confounding effects of combined interventions. The VCS technique may be an advantageous addition to the protocol.

The grafting protocol was more effective in nurse mares introduced to orphan foals than in mares that had rejected their own foals. Approximately half of the rejecting mares altered their behavior and accepted their own foal after hormonal treatment, compared with 76% in the nurse mare group. Foal rejection represents a distinct behavioral and physiological entity, which may be less responsive to hormonally mediated induction. Reports on hormonal treatment for foal rejection cases are limited.^20^^,^^30^^,^^39^ The underlying mechanisms are incompletely understood, but genetic factors and hormonal dysregulation have been suggested because altered progesterone and prolactin expression has been demonstrated in rejecting mares.^40^ Experimental data in rats indicate that disturbances in peripartum oxytocin release may influence maternal behavior.^41^ Arabian horses have been reported to be overrepresented among rejecting mares,^42^ and although there are too few horses to draw any conclusions about breed differences in our dataset, 5 of 6 Arabian mares displayed foal rejection. Of these, 3 were unsuccessful and 2 successful. The Arabian nurse mare had a successful outcome.

In the nurse mare group, younger foal age was the only demographic factor associated with a higher likelihood of successful outcome in our study. This finding is consistent with previous reports suggesting that visual and behavioral stimuli characteristic of the neonatal foal may facilitate maternal acceptance,^43^ and that mare–foal bonding dynamics change as the foal matures.^10^ Foal-related characteristics may play a more prominent role in acceptance success after hormonal grafting than maternal physiological status alone. Although experienced and compliant mares traditionally are preferred for fostering, our results suggest that maternal demographic characteristics alone may not reliably predict treatment success.

The retrospective design of our study limits standardization of drug timing, dosage, and protocol execution. Minor variations in clinical application likely occurred. The multimodal nature of the protocol also prevents isolation of the individual contributions of prostaglandin, oxytocin, and flunixin to treatment outcome. In addition, incomplete data for certain variables and small subgroup sizes, particularly among nulliparous and rejecting mares, may have limited the ability to detect significant associations. However, this case series provides evidence on the efficacy of a hormonal adoption protocol. Although the precise neuroendocrine mechanisms underlying hormonally induced maternal behavior remain incompletely understood, the high immediate response rate observed in our cohort supports the practical clinical utility of the procedure. Based on the dosages and practices observed in our retrospective study, we suggest a protocol that starts with the mare and foal being completely separated. The mare should receive 1.1 mg/kg flunixin meglumine IV, followed by 10 mg dinoprost tromethamine IM after 30 min. When the mare starts sweating, sweat should be collected and used to cover the foal. After an additional 15-20 min, the mare is injected with 20-30 IU oxytocin IV and a few minutes later, the foal is introduced to the mare.

## Conclusions

The grafting protocol described in our study provides a time-efficient, safe, and effective alternative to traditional methods. Considering the decreased need for restraint of the mare, it also has important animal welfare benefits. No adverse effects other than profuse sweating were reported in the treated mares. Future prospective, controlled studies should aim to optimize the protocol through controlled dosage and timing adjustments and elucidate the precise mechanisms of action of the included drugs. Further research into the underlying physiological mechanisms of foal rejection and the lower response to hormonal treatment in some mares is warranted.

## Off-label antimicrobial declaration

The authors declare no off-label use of antimicrobials.

## Institutional animal care and use committee or other approval declaration

The authors declare no institutional animal care and use committee or other approval was needed.

## Abbreviations

NSAID: nonsteroidal anti-inflammatory drug
OR: odds ratio
VCS: vagocervical stimulation
